# Supplementary material for: Generalized Hartmann-Shack array of dielectric metalens sub-arrays for polarimetric beam profiling
Source: Nat Commun. 2018 Nov 2;9:4607. doi: 10.1038/s41467-018-07056-6 (PMC6214988; doi:10.1038/s41467-018-07056-6)
Supplement: Supplementary file 1 — Supplementary Information [file 41467_2018_7056_MOESM1_ESM.pdf]

Supplementary Information for

Generalized Hartmann-Shack array of dielectric metalens sub-arrays for polarimetric beam profiling

Yang et al.

Supplementary Note 1. Details of simulations and design

The characteristics of the unit cell in controlling the amplitude and the phase of the wavefront are analyzed using a finite-difference time-domain (FDTD) approach (Lumerical Inc. FDTD Solutions). In these calculations, the free-space wavelength of the incident light is set to $1550\ \mathrm{nm}$ and the linear polarization axis is parallel to the x-axis. We use periodic boundary conditions along the x- and y-directions and perfectly matched layers (PMLs) along the z-direction. The geometrical parameters are given in the main text.

Each pixel of the metalens array consists of 6 different metalenses, which are designed for “x”, “y”, “a”, “b”, “l”, and “r” polarization. Through the data from Fig. 2b,c and by applying equations (1)-(3), the different metalenses are designed. The result is shown in Supplementary Fig. 1. It serves as the blueprint for the fabricated samples, examples of which are depicted in Fig. 2d-g.

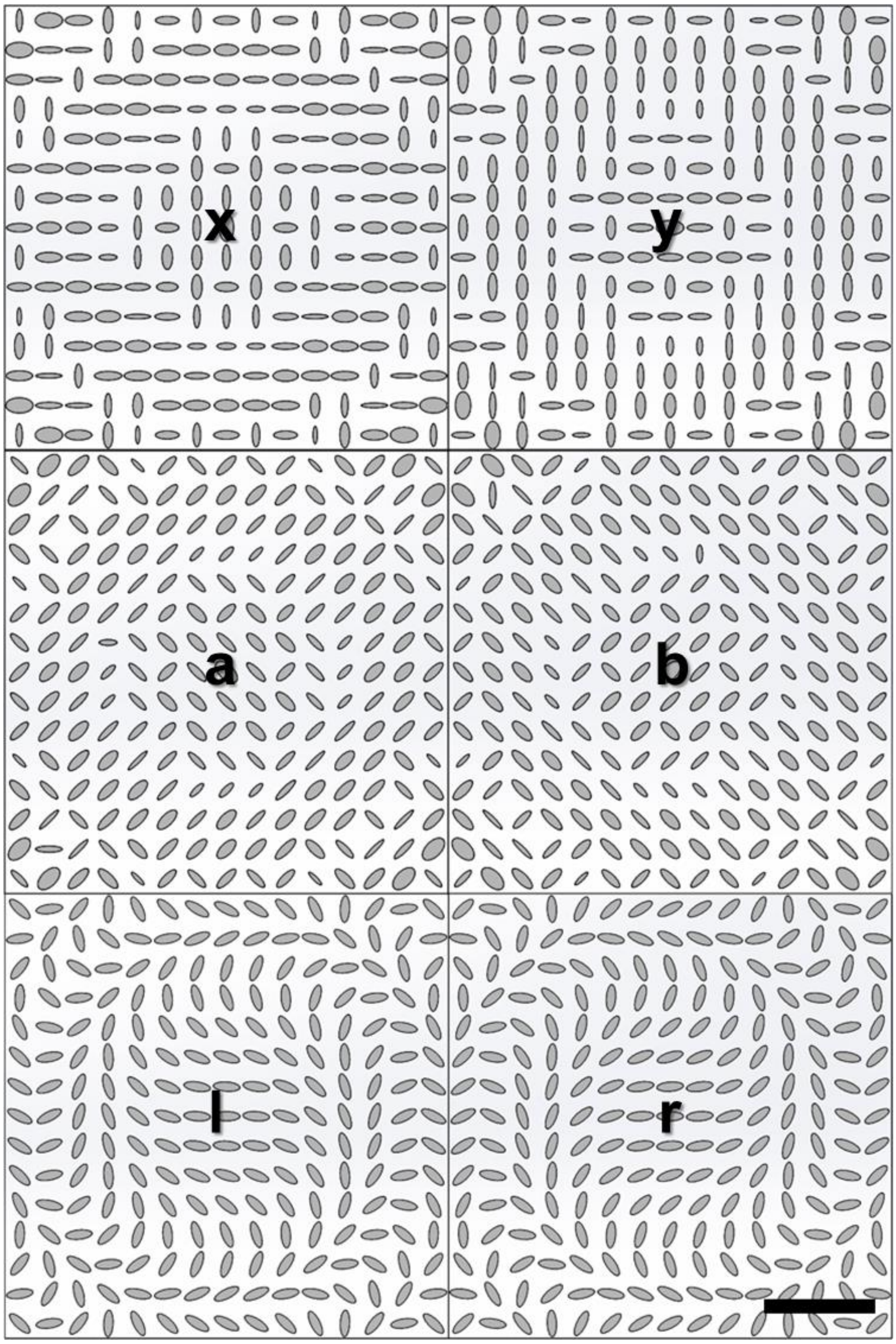

Supplementary Fig. 1: Design of one pixel of the metalens array. Each pixel of the metalens array has six metalenses, one each for “x”, “y”, “a”, “b”, “l”, and “r” polarization. Scale bar: 5 μm.

In order to test and evaluate the actual performance of the designed metalenses, we again use FDTD calculations. Supplementary Fig. 2a-f show the simulated intensity distributions in the metalens focal plane for “x”, “y”, “a”, “b”, “l”, and “r” polarized incident light, respectively.

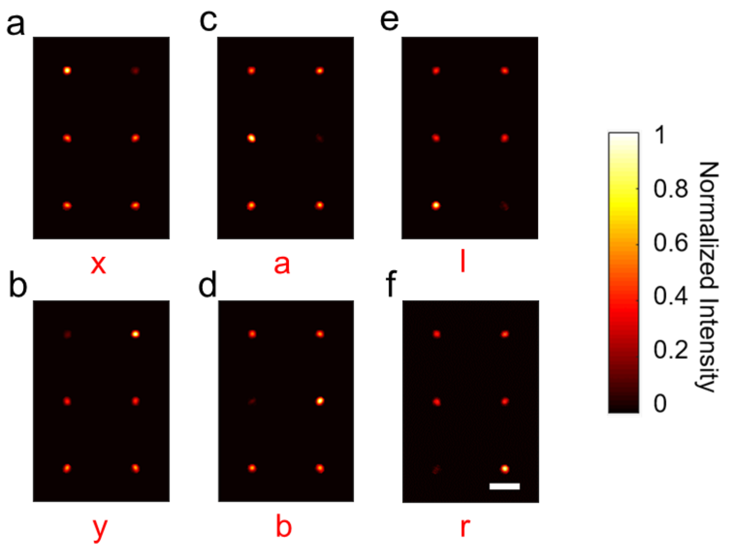

Supplementary Fig. 2: Calculated intensity distributions in the focal plane of one pixel of the metalens array. **a**-**f**, The incident light polarization corresponds to “x”, “y”, “a”, “b”, “l”, and “r” (see main text). Scale bar: 10 μm.

Supplementary Note 2. Details of the fabrication process

Prior to fabricating the metalens array, a double-polished silicon-on-insulator (SOI) wafer is prepared by cleaning. Subsequently, a 430 nm thick ZEP520A electron-beam resist layer is spin-coated onto the wafer and subsequently baked for three minutes on a hot plate at 180 degrees Celsius to complete the curing of the photoresist. Next, to define the patterns, the sample is patterned by electron-beam lithography (EBL, Vistec: EBPG-5000+) at an acceleration voltage of 100 kV and at a beam current of 300 pA, followed by development in xylene and fixation by isopropanol. Afterwards, the sample with the patterned photoresist layers are etched by an inductively-coupled plasma system (Oxford Plasmalab: System100-ICP-180), using C_4F_8/SF_6 gas. The procedure is completed by removal of the patterned residual photoresist in an oxygen plasma (Diener electronic: PICO plasma stripper).

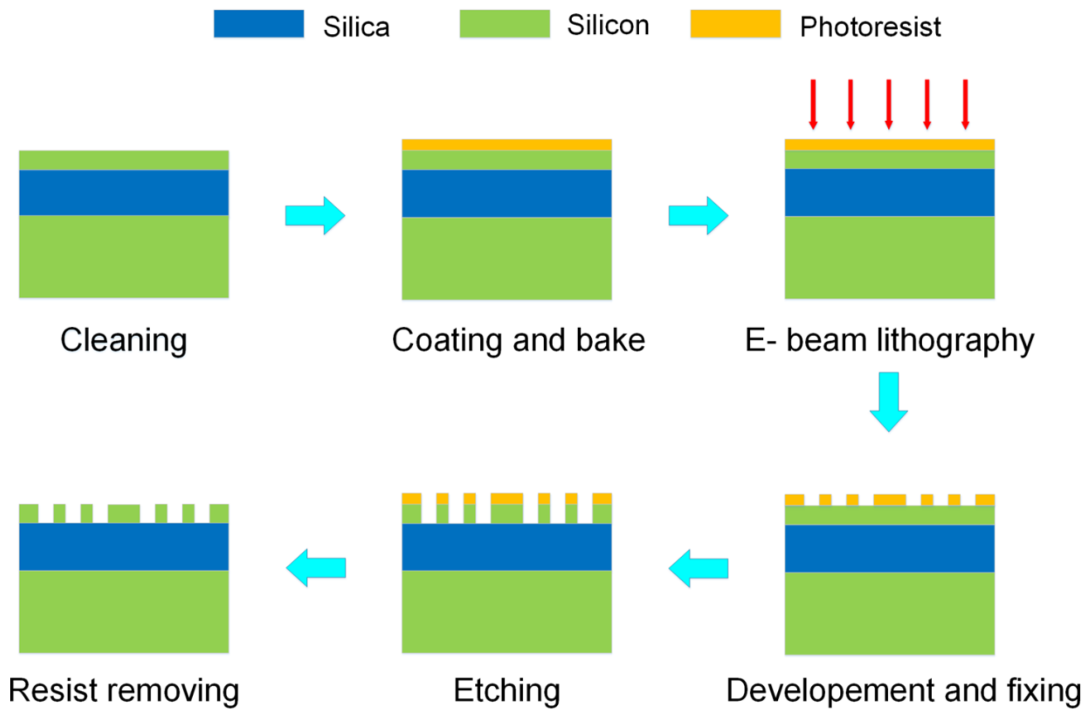

Supplementary Fig. 3: Scheme of the fabrication process of the all-dielectric metalens array.

Supplementary Note 3. Details of the experimental characterization

1. Polarization detections

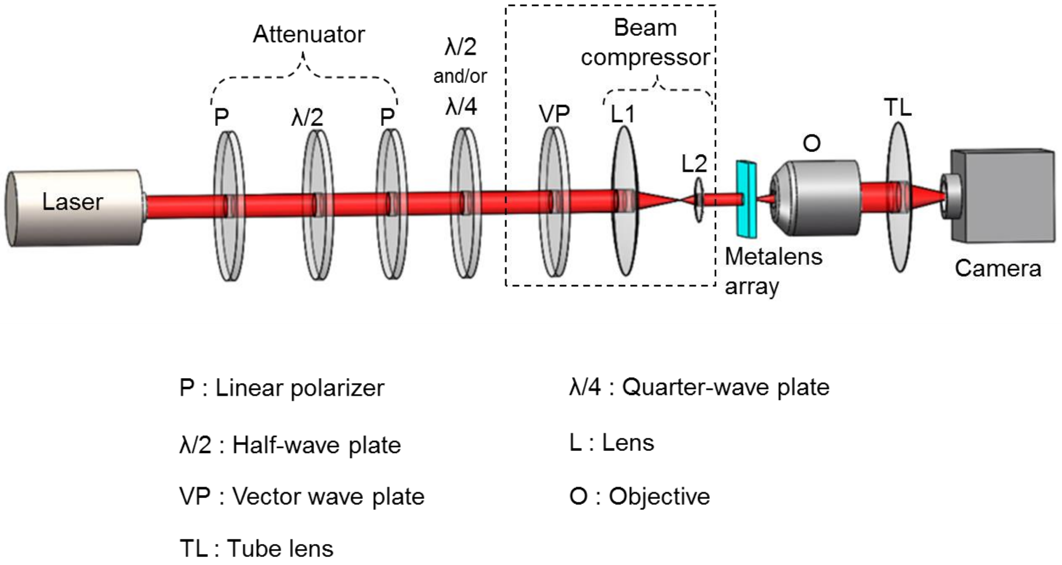

Supplementary Fig. 4: Optical setup for polarimetric beam profiling. The incident beam is derived from a continuous-wave laser (JOINWIT: JW8002) with a free-space wavelength of $1550\,\mathrm{nm}$. The incident power is controlled by an attenuator composed of two linear polarizers and a half-wave plate in between. Thereafter, light beams with 18 different homogeneous polarization states are generated by a combination of half- and/or quarter-wave plates. The emerging light is focused onto the metalens array, and imaged onto the InGaAs-based camera (Xenics: Xeva-1.7-320) by a microscope objective ($20\times$, $\mathrm{NA}=0.5$) and a tube lens. For the measurements of radially polarized and azimuthally polarized beams, respectively, a vector wave plate (Thorlabs: WPV10L-1550) and two lenses, L1 ($200\,\mathrm{mm}$ focal length) and L2 ($50\,\mathrm{mm}$ focal length) are added into the light path (shown in the dashed rectangle).

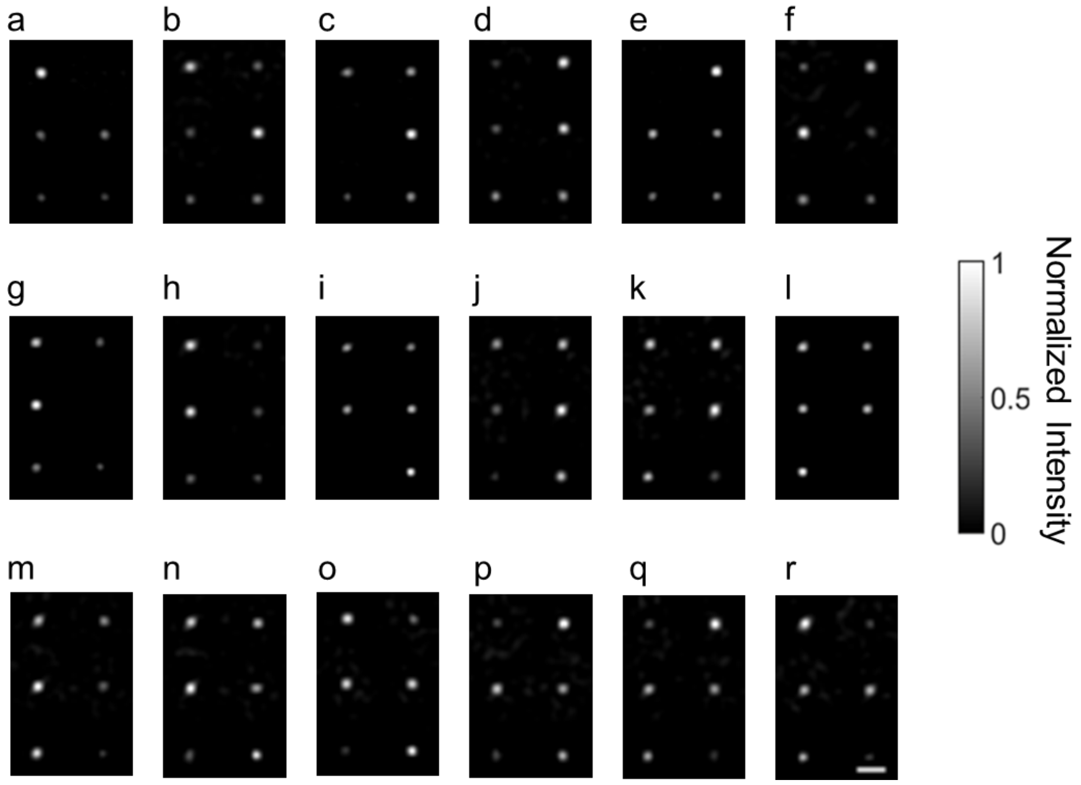

Supplementary Fig. 5: Measurement results (raw data) for different incident polarizations of light. **a-r**, Intensity distributions of one pixel of the metalens array for the 18 different incident polarizations. The extracted parameters are listed in Supplementary Table 1. Scale bar: 10 μm.

Supplementary Table 1.

Theoretical and reconstructed Stokes parameters of the 18 different polarizations.

Orders in Suppl. Fig. 5	Theoretical (S_0, S_1, S_2, S_3)	Reconstructed (S_0, S_1, S_2, S_3)	Relative error
a	(1.000, 1.000, 0.000, 0.000)	(1.000, 1.000, 0.000, 0.000)	0%
b	(1.000, 0.500, -0.866, 0.000)	(1.000, 0.488, -0.873, -0.018)	2.27%
c	(1.000, 0.000, -1.000, 0.000)	(1.000, 0.000, -1.000, 0.000)	0%
d	(1.000, -0.809, -0.588, 0.000)	(1.000, -0.845, -0.534, -0.009)	6.55%
e	(1.000, -1.000, 0.000, 0.000)	(1.000, -1.000, 0.000, 0.000)	0%
f	(1.000, -0.719, 0.695, 0.000)	(1.000, -0.692, 0.722, 0.011)	3.97%
g	(1.000, 0.000, 1.000, 0.000)	(1.000, 0.000, 1.000, 0.000)	0%
h	(1.000, 0.500, 0.866, 0.000)	(1.000, 0.579, 0.813, 0.069)	11.7%
i	(1.000, 0.000, 0.000, 1.000)	(1.000, 0.000, 0.000, 1.000)	0%
j	(1.000, 0.000, -0.707, 0.707)	(1.000, -0.065, -0.660, 0.749)	8.75%
k	(1.000, 0.000, -0.707, -0.707)	(1.000, -0.002, -0.644, -0.765)	8.56%
l	(1.000, 0.000, 0.000, -1.000)	(1.000, 0.000, 0.000, -1.000)	0%
m	(1.000, 0.000, 0.707, -0.707)	(1.000, -0.021, 0.641, -0.764)	9.10%
n	(1.000, 0.000, 0.707, 0.707)	(1.000, -0.026, 0.658, 0.753)	7.20%
o	(1.000, 0.707, 0.000, 0.707)	(1.000, 0.721, -0.033, 0.692)	3.89%
p	(1.000, 0.707, 0.000, -0.707)	(1.000, 0.744, -0.040, 0.667)	6.76%
q	(1.000, -0.707, 0.000, -0.707)	(1.000, -0.648, 0.018, -0.762)	8.26%
r	(1.000, -0.707, 0.000, 0.707)	(1.000, -0.656, 0.055, 0.770)	9.97%
		Average relative error	4.83%

Using the metalens array, 18 different polarized beams are measured. Raw data are shown in Supplementary Fig. 5. The intensity of a certain polarization measured in this work is an integral over a square area of the CCD camera. The square area basically

covers the entire focusing spot, which is about 5×5 pixels of the CCD camera. The projection of one metalens covers 16×16 pixels on the CCD. The theoretical and measured Stokes parameters are compared in Supplementary Table 1. The definition of the relative error is the modulus of the difference between theoretical and measured points on the Poincare sphere divided by the sphere's radius.

2. Phase detection of a vortex-beam

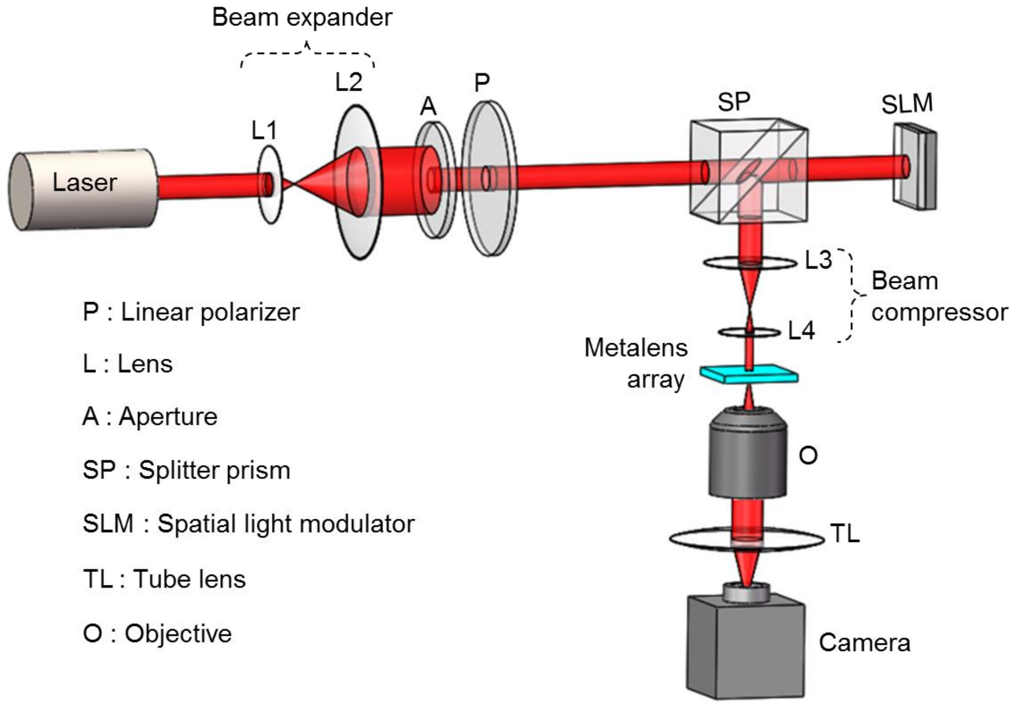

Supplementary Fig. 6: Optical setup for the vortex-beam profiling. The beam expander, composed of two lenses, L1 (50 mm focal length) and L2 (200 mm focal length, forms the laser beam with the plane wavefront. An aperture reduces the beam diameter to 8 mm. A linear polarizer and a polarizing beam splitter are placed behind the aperture. A spatial light modulator generating the vortex beam follows. The vortex beam is compressed by two lenses, L3 (300 mm focal length) and L4 (40 mm focal length) to exclusively illuminate the metalens array. Finally, the light passes through a microscope objective lens ($20\times$, $\mathrm{NA} = 0.5$) and a tube lens to arrive at the image on the InGaAs-based camera.

3. Simultaneous detection of polarization and phase

In order to demonstrate that the system does have the ability of simultaneous

detection of polarization and phase, we carried out the following experiment.

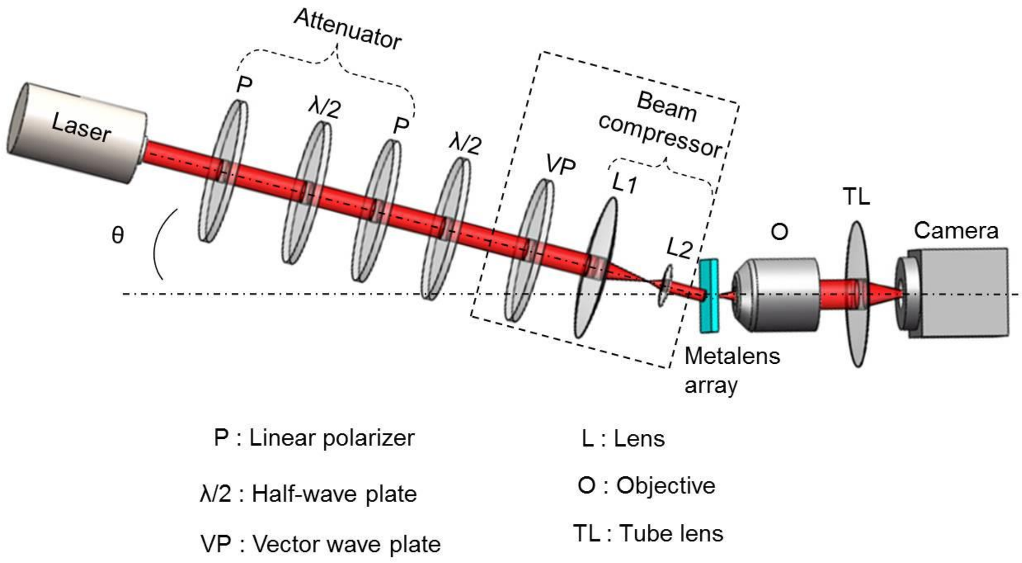

Supplementary Fig. 7: Optical setup for an oblique incident radially polarized beam profiling. All experimental parameters are the same as in Supplementary Fig. 4 except for the incidence angle (θ) of 7.4°.

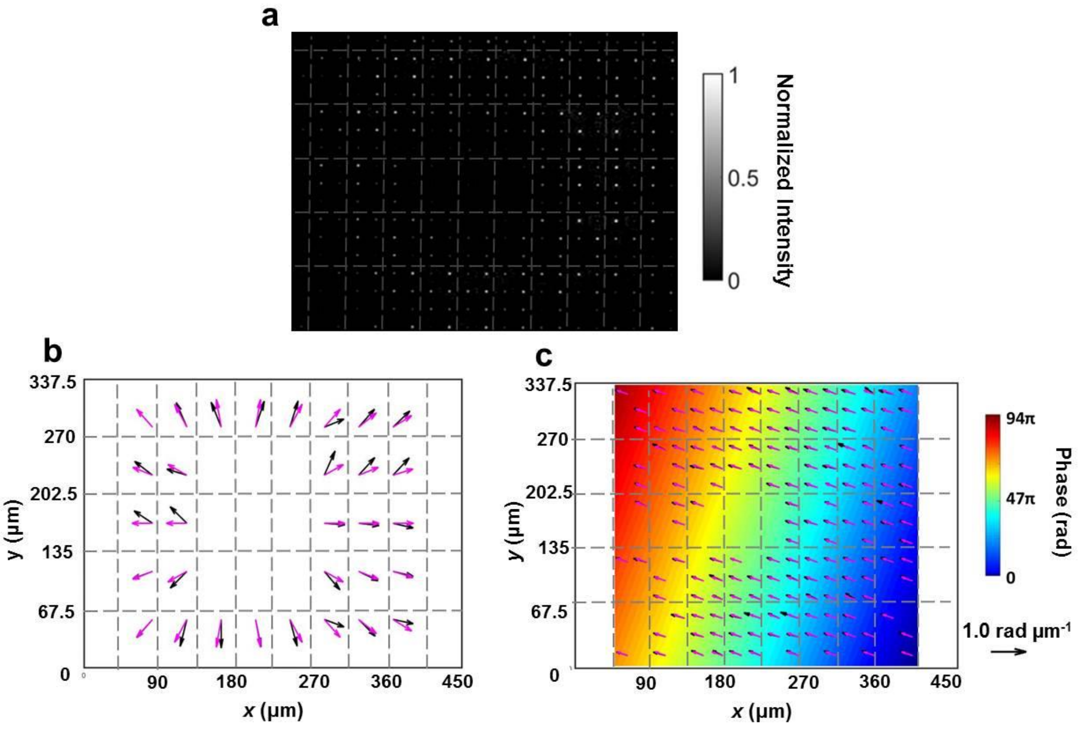

Supplementary Fig. 8: Profiling of an oblique incident radially polarized beam by the metalens array. **a**, Raw data of measured focal spots of the metalens array. **b**, Polarization profile. The black arrows correspond to the measured local polarization vectors, the pink arrows to the calculated ones. **c**, Phase

gradients (black arrows for the measured, pink arrows for the calculated) and wave front (false-colour scale) reconstructed on this basis. The dashed lines highlight the individual pixels of the 10×5 metalens array. The reference arrow has a length of 1.0 rad μm^{-1}

4. Detection of degree of polarization (DOP)

In order to demonstrate that the system can also work for partially polarized beams, we carried out the following experiment.

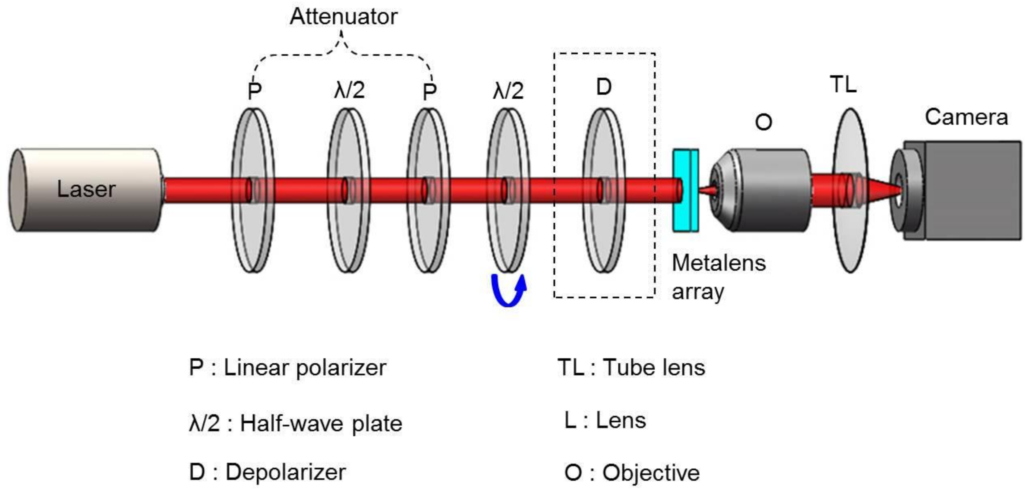

Supplementary Fig. 9: Optical setup for measurement of degree of polarization (DOP). The incident beam is derived from a continuous-wave laser (JOINWIT: JW8002) with a free-space wavelength of 1550 nm. The incident power is controlled by an attenuator composed of two linear polarizers and a half-wave plate in between. Thereafter, linearly polarized light beams with different polarization angles are generated by a half-wave plate. In order to produce partially polarized light, a depolarizer (Union Optic: QWD3225, shown in the dashed rectangle) is put in the setup, which is quartz-quartz wedge depolarizer. When the polarization angle of the incident light changes, the DOP of the output light changes. The emerging light impinges onto the metalens array, and is imaged onto the InGaAs-based camera (Xenics: Xeva-1.7-320) by a microscope objective ($20\times$, NA $= 0.5$) via a tube lens.

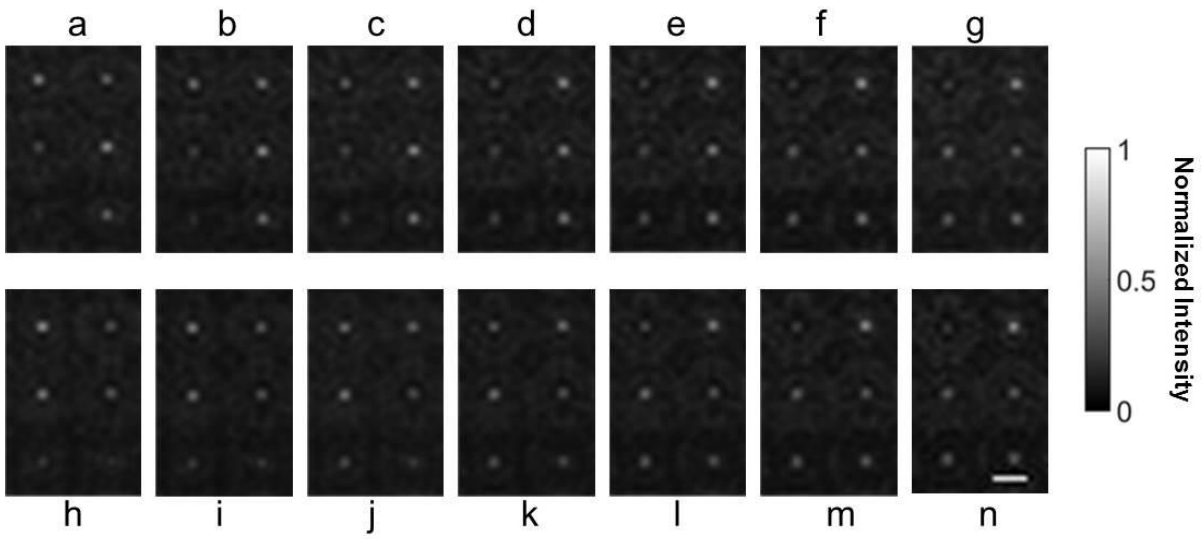

Supplementary Fig. 10: Measurement results (raw data) without/with the depolarizer for different incident polarization angles of light. **a-g**, Intensity distributions of one pixel of the metalens array for incident polarization angles from -30 ° to -90 ° without the depolarizer. **h-n**, Results with the depolarizer. Scale bar: 10 µm.

Supplementary Table 2.

Calculated DOPs based on the raw data in Supplementary Fig. 10.

	Orders in Fig. 2	Polarization angles*	DOP+		Orders in Fig. 2	Polarization angles*	DOP+
Without depolarizer	a	-30°	99.03%	With depolarizer	h	-30°	97.26%
	b	-40°	91.93%		i	-40°	91.41%
	c	-50°	98.14%		j	-50°	68.67%
	d	-60°	94.29%		k	-60°	49.09%
	e	-70°	98.42%		l	-70°	65.84%
	f	-80°	93.36%		m	-80°	83.15%
	g	-90°	99.99%		n	-90°	92.54%

*: Polarization angles of the light through the half-wave plate

+: Degree of polarization

Supplementary Note 4. Details of the algorithm for reconstruction of Stokes parameters

Ideally, the amplitude of a monochromatic plane wave propagating along the z-direction can be defined (in Cartesian coordinates) by the Jones vector

$$\boldsymbol{E}_0 = (A_\mathrm{x}\,, \quad A_\mathrm{y} e^{i\delta})^\mathrm{T}\,, \tag{1}$$

where A_x and A_y are the amplitudes along x- and y- direction, respectively, and δ is the phase difference between the two components. The Stokes parameters follow:

$$s_0 = A_\mathrm{x}^2 + A_\mathrm{y}^2\,, \tag{2}$$

$$s_1 = A_\mathrm{x}^2 - A_\mathrm{y}^2\,, \tag{3}$$

$$s_2 = 2A_\mathrm{x}A_\mathrm{y}\cos\delta = A_\mathrm{a}^2 - A_\mathrm{b}^2, \tag{4}$$

$$s_3 = 2A_\mathrm{x}A_\mathrm{y}\sin\delta = A_\mathrm{r}^2 - A_\mathrm{l}^2\,, \tag{5}$$

It is clear that s_0 is proportional to the intensity of the beam, whereas s_1, s_2, s_3 describe the state of polarization. Obviously, there is a linear relationship between the Stokes parameters and the six intensities $(I_\mathrm{x}, I_\mathrm{y}, I_\mathrm{a}, I_\mathrm{b}, I_\mathrm{l}, I_\mathrm{r})$, which is described by a 4×6 matrix $\boldsymbol{M}$ given by

$$\begin{pmatrix} s_0 \\ s_1 \\ s_2 \\ s_3 \end{pmatrix} = \boldsymbol{M} \cdot \begin{pmatrix} I_\mathrm{x} \\ I_\mathrm{y} \\ I_\mathrm{a} \\ I_\mathrm{b} \\ I_\mathrm{l} \\ I_\mathrm{r} \end{pmatrix} = \begin{pmatrix} 1 & 1 & 0 & 0 & 0 & 0 \\ 1 & -1 & 0 & 0 & 0 & 0 \\ 0 & 0 & 1 & -1 & 0 & 0 \\ 0 & 0 & 0 & 0 & -1 & 1 \end{pmatrix} \cdot \begin{pmatrix} I_\mathrm{x} \\ I_\mathrm{y} \\ I_\mathrm{a} \\ I_\mathrm{b} \\ I_\mathrm{l} \\ I_\mathrm{r} \end{pmatrix}, \tag{6}$$

where (s_0, s_1, s_2, s_3) are the Stokes parameters and $(I_\mathrm{x}, I_\mathrm{y}, I_\mathrm{a}, I_\mathrm{b}, I_\mathrm{l}, I_\mathrm{r})$ the intensities of six polarization components.

However, equation (6) applies only for the ideal situation. In the actual experiment, the measured focal spot intensities behind the device are generally disturbed by background. This background consists of light transmitted directly through the substrate

as well as of other polarization components which are not modulated by the metasurfaces. We assume a linear relationship between the background intensities $(B_\mathrm{x}, B_\mathrm{y}, B_\mathrm{a}, B_\mathrm{b}, B_\mathrm{l}, B_\mathrm{r})$ contained in the six focusing spots and the intensities of the six polarization components $\boldsymbol{I}_0$

$$\boldsymbol{B} = \boldsymbol{M}_1 \cdot \boldsymbol{I}_0\,, \tag{7}$$

where $\boldsymbol{B} = (B_\mathrm{x}, B_\mathrm{y}, B_\mathrm{a}, B_\mathrm{b}, B_\mathrm{l}, B_\mathrm{r})^\mathrm{T}$ are the intensities of the background beam, $\boldsymbol{I_0} = (I_\mathrm{x}, I_\mathrm{y}, I_\mathrm{a}, I_\mathrm{b}, I_\mathrm{l}, I_\mathrm{r})^\mathrm{T}$ are the effective intensities of the focusing spots, and $\boldsymbol{M_1}$ is a 6×6 matrix. Moreover, the relationship between $\boldsymbol{I_0}$ and the measured intensities $\boldsymbol{I}$ can be described as follows:

$$\boldsymbol{I} - \boldsymbol{B} = \boldsymbol{M}_2 \cdot \boldsymbol{I}_0\,, \tag{8}$$

where $\boldsymbol{I}$ describes the intensities of the six focusing spots measured in the experiment, and $\boldsymbol{M}_2 = diag(t_\mathrm{x}, t_\mathrm{y}, t_\mathrm{a}, t_\mathrm{b}, t_\mathrm{l}, t_\mathrm{r})$ is a diagonal matrix whose values are related with the focusing ability of the six metalenses. Therefore, combining the equations (6)-(8), the relationship between the Stokes parameters $\boldsymbol{S}$ and the measured intensities $\boldsymbol{I}$ can be described as follows:

$$\boldsymbol{S} = \boldsymbol{M} \cdot \boldsymbol{I}_0 = \boldsymbol{M} \cdot (\boldsymbol{M}_1 + \boldsymbol{M}_2)^{-1} \cdot \boldsymbol{I} = \boldsymbol{A} \cdot \boldsymbol{I} \tag{9}$$

Here, $\boldsymbol{A}$ is a 4×6 ultimate reconstructed matrix. To calculate the matrix $\boldsymbol{A}$, we choose six special incident polarizations (x-, y-, a-, b-, l-, r- polarization) and measure the intensities of the focusing spots, respectively. In this manner, the reconstructed matrix $\boldsymbol{A}$ can be calculated by using the equation $\boldsymbol{A} = \boldsymbol{S} \cdot \mathrm{pinv}(\boldsymbol{I})$.
